# Fluticasone furoate/fluticasone propionate – different drugs with different properties

**DOI:** 10.1111/j.1752-699X.2011.00244.x

**Published:** 2011-07

**Authors:** Keith Biggadike

**Affiliations:** Allergic Inflammation DPU, Respiratory CEDD, GlaxoSmithKline Medicines Research CentreStevenage, Hertfordshire, UK

The similarity in the names of the recently introduced intranasal glucocorticoid fluticasone furoate (FF; Veramyst®, GlaxoSmithKline/Avamys®, GlaxoSmithKline UK, Uxbridge, UK) and the earlier fluticasone propionate (FP; Flonase®/Flixonase®, GlaxoSmithKline) has led many to assume that the two compounds have the same active principle (fluticasone) (e.g. 1, 2). This has been compounded by FP commonly, and incorrectly, being abbreviated to fluticasone. The purpose of this letter is to highlight that FF and FP are completely different drugs with FF showing distinct and superior properties (3), and hence prevent any misprescription of these drugs in the future.

**Figure 1:**
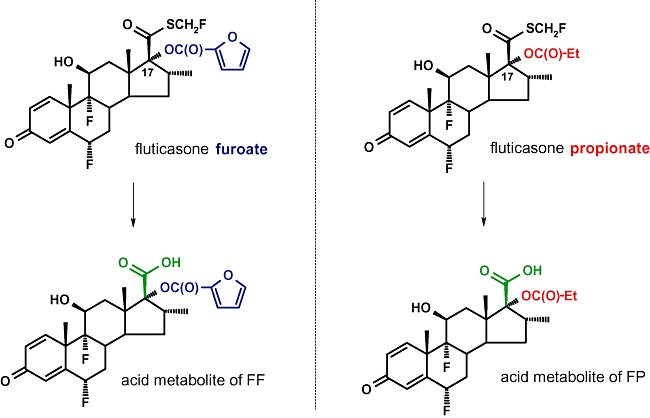
Structures of fluticasone furoate and fluticasone propionate and their major metabolites.

This confusion clearly stems from the unusual assigned glucocorticoid nomenclature which splits these molecules into the steroidal backbone (fluticasone) and the ester substituent (furoate/propionate). This naming convention does suggest that these derivatives could be ester prodrugs of fluticasone. In fact, a number of topical glucocorticoid esters are indeed ester prodrugs releasing the active parent glucocorticoid in the body. However, fluticasone 17α esters are remarkably stable and remain attached to the fluticasone backbone even during metabolism. Their pharmacological activity is mediated by the entire molecule (backbone + ester) and they share no common metabolites (Fig. 1) – neither FF nor FP is metabolised to fluticasone. FF and FP are therefore structurally distinct drug substances with distinct properties.

The furoate and propionate moieties are far from inert appendages but serve to significantly enhance the glucocorticoid activity of fluticasone, which has never itself been developed. Key interactions of FF with the glucocorticoid receptor have been elucidated by X-ray crystallography which shows the ester derived from 2-furoic acid occupying a discrete pocket on the receptor much more completely than does the smaller propionate ester of FP (4). The resulting enhanced affinity of FF for the target receptor is reflected in the lower daily dose of Veramyst (110 µg) compared with Flonase (200 µg).

The ester group also contributes to the physicochemical characteristics of the molecule which impact on solubility, dissolution rate, tissue affinity, and hence pharmacokinetic and pharmacodynamic properties. Thus, the ester derived from 2-furoic acid in FF confers higher affinity for both nasal and lung tissue compared with FP (5, 6) and recent studies with inhaled FF have shown that this translates to enhanced lung residency and once-daily efficacy in asthma (7, 8). There is already some evidence that the characteristics of FF may result in superior symptom reduction compared with FP (9, 10) or similar improvements in symptoms at less frequent dosing schedules (11), which could result in reduced health-care costs/concomitant medication use (12); however, prospective, randomised, head-to-head studies are required to provide a definitive answer. With new inhaled products containing FF in Phase III trials (Relovair®, GlaxoSmithKline) it is important for prescribers to understand that this is a novel glucocorticoid, not to be confused with FP. Moreover, the practice of abbreviating FP and FF to fluticasone should be discouraged.
